# Value of Dedicated Head and Neck ^18^F-FDG PET/CT Protocol in Detecting Recurrent and Metastatic Lesions in Post-surgical Differentiated Thyroid Carcinoma Patients with High Serum Thyroglobulin Level and Negative ^131^I Whole-body Scan

**DOI:** 10.7508/aojnmb.2016.04.003

**Published:** 2016

**Authors:** Mai Hong Son, Bui Quang Bieu, Le Ngoc Ha

**Affiliations:** Department of Nuclear Medicine, Tran Hung Dao Hospital, Hanoi, Vietnam

**Keywords:** ^18^F-FDG PET/CT, Differentiated Thyroid Carcinoma, Head and Neck, Thyroglobulin

## Abstract

**Objective(s):**

In clinical practice, approximately 10-25% of post-surgical differentiated thyroid carcinoma (DTC) patients with high serum thyroglobulin (Tg) and negative ^131^I whole-body scan (WBS) have poor prognosis due to recurrent or metastatic lesions after radioactive iodine treatment. The purpose of this study was to evaluate the value of ^18^F-FDG PET/CT scan in DTC patients with high serum Tg level and negative ^131^I WBS.

**Methods:**

69 post-surgical DTC patients with high serum Tg level and negative post ablation ^131^I WBS were enrolled in this study. All DTC patients underwent head and neck ultrasound, CT scan and whole-body ^18^F-FDG PET/CT, based on the dedicated head and neck protocol.

**Results:**

Overall, 92 lesions were detected in 43 (62.3%) out of 69 patients with positive ^18^F-FDG PET/CT scan, compared to only 39 lesions detected on CT scan in 26 (37.7%) out of 69 patients. The sensitivity, accuracy and negative predictive value of ^18^F-FDG PET/CT were 88%, 87% % and 76%, respectively, which were significantly higher than those of CT scan (67.2%, 54.3% and 48.8%, respectively) (P<0.01). Specificity and positive predictive value of ^18^F-FDG PET/CT (90.5% and 95.2%, respectively) were similar to those of CT scan (95.2% and 96.2%, respectively) (P>0.05). The maximum standardized uptake value (SUV_max_) threshold was 4.5 with a good diagnostic value (sensitivity of 92.3% and specificity of 100%). The dedicated head and neck ^18^F-FDG PET/CT protocol altered the treatment plan in 33 (47.8%) out of 69 DTC patients with high serum Tg level and negative ^131^I WBS.

**Conclusion:**

Dedicated head and neck ^18^F-FDG PET/CT protocol showed a higher diagnostic value, compared to CT scan and played an important role in detecting recurrent or metastatic lesions in post-surgical DTC patients with high serum Tg level and negative ^131^I WBS.

## Introduction

In Vietnam, thyroid cancer is the sixth most common malignant disease, following lung, breast, prostate, cervical and hepatocellular carcinomas in both men and women (1). Differentiated thyroid carcinoma (DTC), which accounts for 90% of all thyroid cancers, often has a good prognosis due to radioiodine avidity. However, the rate of recurrence and metastasis remains high in high-risk DTC patients. Overall, 10-25% of patients with DTC may experience tumor recurrence and/or metastasis in the course of their life (2).

Traditionally, after total thyroidectomy and remnant ablation with radioactive iodine (RAI), DTC patients are followed-up to detect recurrence and metastasis by serum thyroglobulin (Tg) and diagnostic iodine-131 (^131^I) whole-body scan (WBS). Determination of abnormalities in serum Tg level and ^131^I WBS plays a significant role in clinical decision-making by nuclear medicine physicians.

According to our statistical data, 15-20% of DTC patients have high serum Tg level, despite negative ^131^I WBS (positive Tg and negative ^131^I WBS) after ^131^I therapeutic courses (3). Lack of response to RAI therapy is predicted in these patients, who have a poorer prognosis, compared to others. In case ^131^I WBS cannot detect recurrent or metastatic lesions in DTC patients with high serum Tg level, other diagnostic imaging modalities such as computed tomography (CT), neck ultrasound, magnetic resonance imaging (MRI) and fluorine-18 fluorodeoxyglucose (^18^F-FDG) positron emission tomography (PET)/CT are recommended as complementary methods (4). Findings obtained by conventional imaging modalities (e.g., ultrasound, CT and MRI) are often equivocal in many cases with post-surgical structural changes.

Nowadays, ^18^F-FDG PET/CT by fusing anatomic and molecular imaging is considered as an effective modality in many malignant diseases (5). Several studies have confirmed the value of whole-body ^18^F-FDG PET/CT scan in DTC patients with positive Tg and negative ^131^I WBS, despite its limitations in post-surgical examination of head and neck regions (6). However, to the best of our knowledge, a limited number of studies have evaluated the value of ^18^F-FDG PET/CT in DTC patients in Vietnam.

In our center, dedicated head and neck ^18^F-FDG PET/CT protocol has been proposed for routine application in order to increase the diagnostic accuracy. Therefore, the main purpose of this study was to evaluate the utility of dedicated head and neck ^18^F-FDG PET/CT protocol in the diagnosis of DTC patients with positive Tg and negative ^131^I WBS.

## Methods

In the present study, 69 DTC patients with negative post-therapeutic ^131^I WBS and high Tg level (> 2 ng/ml) after thyroid hormone withdrawal associated with normal anti-Tg level, were selected at the Department of Nuclear Medicine, Tran Hung Dao Hospital, Hanoi, Vietnam during April 2010-August 2013. Informed consent forms were signed by all participants, as required by the medical ethics committee of the hospital.

Patients underwent ^18^F-FDG PET/CT scan, using the dedicated head and neck protocol. The diagnostic values of CT and ^18^F-FDG PET/CT were analyzed, based on relevant pathologic and histologic results and follow up(every six-months). PET/CT examination was performed, using GE Discovery Lightspeed 16-slice CT scan (STE), according to the European Association of Nuclear Medicine (EANM) guidelines, version 1.0 (7).

For patient preparation, the serum glucose level was checked to exclude hyperglycemia. Afterwards, the patients were allowed to rest in the waiting room before intravenous injection of 2.5 MBq/kg body weight (±10%) of ^18^F-FDG. PET and low-dose CT scans from the skull base to the mid-thigh were performed 60 min after ^18^F-FDG injection as the standard procedure.

Subsequent to the whole body PET/CT imaging, the dedicated head and neck protocol was applied. If the head and neck diagnostic CT scan with contrast enhancement was not available within four weeks prior to ^18^F-FDG PET/CT scan, the dedicated head and neck ^18^F-FDG PET and CT scan, using an intravenous contrast, were performed.

The dedicated head and neck protocol was acquired from the cranial top to the thoracic inlet in an arm-down position. Briefly, PET images were obtained in a three-dimensional mode, two bed positions (for 6 min per bed position), 30 cm transaxial field of view (FOV), iterative reconstruction, 20 subsets/2 iterations and a matrix size of 256×256.

Attenuation correction was based on contrast-enhanced CT scan (if present). The parameters of head and neck CT scan were as follows: 120 kVp, 100 mA, helical thickness of 3.75 mm, matrix size of 512×512, FOV of 30 cm and 0.5 s/rotation. Overall, 100 ml of the contrast material was used (if necessary) with a scan delay of 30 s and an injection rate of 3 ml/s (8). Tumor-node-metastasis (TNM) staging was used for risk stratification, according to the classification by the American Joint Committee on Cancer (AJCC) (9).

In general, ^18^F-FDG PET/CT was read independently by one of two nuclear medicine physicians with thorough knowledge about the patient’s clinical history; then, the other physician reviewed all lesion-related findings and impressions. Disagreements were resolved by consensus. A positive lesion on F-18 FDG PET/CT imaging was defined as a focal FDG uptake with relatively higher activity than that of the surrounding normal tissue or when the SUV_max_ of the lesion was revealed to be more than 2.5.

All detected lymph nodes were measured and a threshold of 10 mm was considered for pathologic lymph nodes (10). The diagnosis of recurrent and metastatic lesions was confirmed by histopathologic results or follow-up for at least 12 months. The diagnosis was confirmed by elevation in serum Tg level (≥10 ng/ml after T_4_ withdrawal) or other imaging modalities such as neck ultrasonography and CT scan.

The statistical analysis was performed, using SPSS version 16.0 (SPSS Inc, Chicago, IL). The sensitivity, specificity, positive and negative predictive values and accuracy of ^18^F-FDG PET/CT and CT scan were analyzed, and the confidence intervals (95%) were determined.

## Results

In total, 69 DTC patients including 13 males (18.8%) and 56 females (81.2%), with negative ^131^I WBS and high serum Tg level, were admitted to our center for dedicated head and neck ^18^F-FDG PET/CT examination (Table 1).

**Table 1:** Clinical characteristics of DTC patients in the study

Characteristics	Value (n, %)
Total	69 (100%)
Male	13 (18.8%)
Female	56 (81.2%)
Age (year)	
Range	20 – 80
Mean±SD	45.6±13.2
Histologic subtypes	
Papillary thyroid carcinoma	66 (95.6%)
Follicular thyroid carcinoma	3 (4.4%)
TNM staging	
Stage I	26 (37.7%)
Stage II	10 (14.5%)
Stage III	3 (4.3%)
Stage IV	17 (24.6%)
Unidentified	13 (18.8%)
Number of I-131 therapy course	
Range	2 - 10
Mean±SD	7.8±5.3
Total ^131^I dose (mCi)	
Range	100 – 800
Mean±SD	468.6 ± 271.3
Tg with thyroid hormone withdrawal (ng/ml)	
Range	11.4 – 1000
Mean±SD	218.3 ± 262.1
Number of patients with recurrence/metastatic thyroid carcinoma	
Postoperative positive histopathology	32
Follow – up positive images and/or Tg	13
Time of Follow – up (month)	
Range	12 - 43
Mean±SD	24.1±9

**Figure 1:**
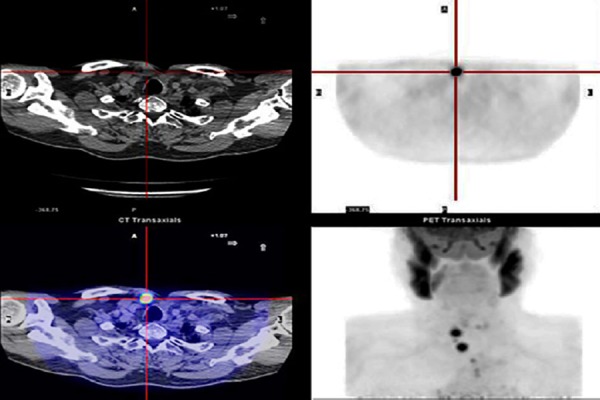
Images of a 65-year-old male patient with papillary thyroid cancer undergoing total thyroidectomy and ^131^I therapy with a total dose of 550 mCi. ^131^I WBS after the third treatment session showed no abnormal uptake and the patient’s stimulated thyroglobulin was > 1000 ng/ml. (B) axial PET and (C) axial PET/CT images revealed increased focal ^18^F-FDG uptake in thyroid bed and pretracheal lymph node, which is not clearly seen in low-dose CT (A). Surgical resection of these lesions confirmed recurrence/metastasis of papillary thyroid carcinoma

**Figure 2:**
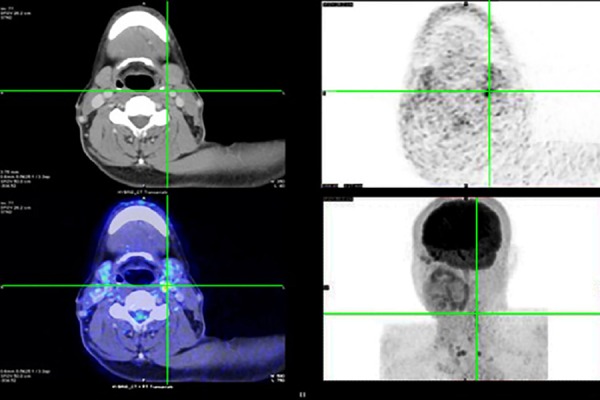
Dedicated head and neck ^18^F-FDG PET/CT with contrast enhancement in a 60-year-old male patient with a prior history of thyroid papillary carcinoma and total thyroidectomy. The patient presented with a serum thyroglobulin level of 1000 ng/ml and negative post-therapeutic ^131^I WBS. The axial PET image illustrated focal ^18^F-FDG uptake (SUV_max_: 7.6), correlated with the sub-centimeter lymph node on the left IIA level. ^18^F-FDG PET/CT with contrast enhancement precisely localized the lesion which may be confused as normal on CT imaging alone. After resection, the histopathological results indicated metastatic lymph node of thyroid carcinoma

**Figure 3:**
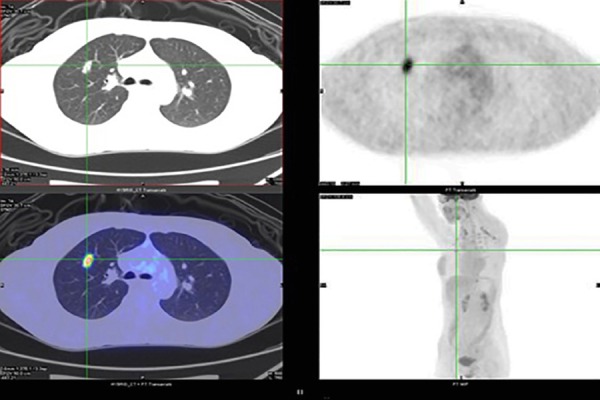
Whole-body ^18^F-FDG PET/CT scan of a 32-year-old female patient with a prior history of papillary thyroid carcinoma undergoing total thyroidectomy and negative ^131^I WBS. The patient presented with a serum thyroglobulin level of 862 ng/ml. Axial low-dose CT and ^18^F-FDG PET/CT demonstrated a small solitary pulmonary nodule (10×15 mm in diameter) and increased ^18^F-FDG avidity (SUV_max_: 7.5) on the right upper lobe. Metastatic lesion was histologically confirmed by transthoracic biopsy

The characteristics of lesions on CT and ^18^F-FDG PET/CT scans are presented in Table 2. In total, 92 lesions were detected in 43 patients (62.3%) on ^18^F-FDG PET/CT, compared to only 39 lesions detected in 26 patients (37.7%) on CT scans. ^18^F-FDG PET/CT could detect more malignant lesions in thyroid beds, cervical lymph nodes and distant metastases, compared to CT scan alone (Figures 1, 2 & 3).

**Table 2:** Lesion characteristics in CT and ^18^F-FDG PET/CT scans

Characteristics		CT	PET/CT
Number of patients with positive findings		26 (37.7%)	43 (62.3%)
Number of lesions		39	92
Lesion size (mm)	Minimum	5	4
	Maximum	27	27
	Mean±SD	12.8±4.3	9.6±4.6
SUV_max_ (g/ml)	Minimum		3.0
	Maximum		28
	Mean±SD		7.8±5.3
Lesion site	Thyroid bed	10	20
	Cervical lymph node	20	55
	Mediastinal lymph node	4	12
	Lung	5	5

The sensitivity, accuracy and negative predictive value of ^18^F-FDG PET/CT (87%, 88% and 76%, respectively) were significantly higher than those of CT scan alone (54.3%, 67.2% and 48.8%, respectively) (P<0.01). The specificity and positive predictive value of both CT and ^18^F-FDG PET/CT scans were similar (95.2% and 96.2% for CT scan and 90.5% and 95.2% for ^18^F-FDG PET/CT, respectively) (P>0.05).

**Figure 4:**
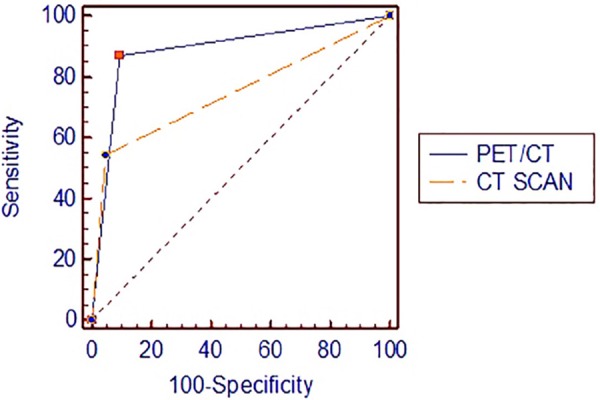
Comparison of ROC curves of CT and ^18^F-FDG PET/CT scans in the diagnosis of recurrent/metastatic lesions in DTC patients

The area under the receiver operating characteristic (ROC) curve of ^18^F-FDG PET/CT (0.887) was significantly larger than that of CT scan (0.748), as presented in Figure 4. The overall results indicated that ^18^F-FDG PET/CT examination is superior to CT scan in the diagnosis of recurrent and/or metastatic DTC.

Based on positive ^18^F-FDG PET/CT findings, the treatment strategy directly changed in 33 (47.8%) out of 69 DTC patients. Particularly, 31 (86%) out of 33 patients required further surgeries and 2 (4%) out of 33 patients were refferred for external beam radiation therapy. The empiric ^131^I therapy was performed on 27 (39%) out of 69 cases. Also, in 9 (13.2%) out of 69 patients with negative FDG-PET/CT imaging, further treatment was not justified, and this group was pursued to follow the watchful waiting strategy (Table 3).

**Table 3:** Therapeutic changes after the implematation of ^18^F-FDG PET/CT

Treatment plan	n	%
Surgery	31	44.9
External beam radiation therapy (EBRT)	2	2.9
Empirical ^131^I therapy	27	39.1
Watchful waiting strategy	9	13.1

## Discussion

The present study demonstrated that dedicated head and neck ^18^F-FDG PET/CT scan is a promising modality for detecting and localizing local recurrences, regional metastatic lymph nodes and distant metastases in DTC patients with positive Tg and negative ^131^I WBS. Moreover, this modality had a potential impact on physicians’ decision-making.

In this regard, as Bannas at al. indicated, the overall sensitivity, specificity and accuracy of ^18^F-FDG PET/CT were 68%, 60% and 66.7%, respectively in 30 DTC patients with negative ^131^I WBS and high serum Tg level (11). Moreover, Shammas et al. in a study on 61 patients revealed the sensitivity, specificity and accuracy of ^18^F-FDG PET/CT to be 68.4%, 82.4% and 73.8%, respectively for the diagnosis of recurrence or metastasis (12). However, these values seem to be lower than the presented data in our study, which might be due to the smaller number of patients and differences in the selected patient populations in studies by Bannas et al. and Shammas and colleagues.

As confirmed by other studies, the sensitivity of ^18^F-FDG PET in DTC patients with negative I-131 WBS and high serum Tg level is also dependent on ^18^F-FDG uptake (13) (14). Generally, the anatomical landmarks and post-operative structures of the head and neck regions may be confusing for radiologists and nuclear medicine physicians in interpreting ^18^F-FDG PET/CT images. Several earlier studies using dedicated head and neck ^18^F-FDG PET/CT protocol showed that the sensitivity, specificity and accuracy of this modality may be improved compared to conventional method in order to assure nuclear medicine physicians about the analysis of structural complexity of head and neck regions (15,16).

In our study, the dedicated head and neck ^18^F-FDG PET/CT protocol and contrast enhancement could provide better results, compared to the routine whole-body ^18^F-FDG PET/CT scan in detecting and localizing regional recurrences and/or metastatic lesions in post-surgical DTC patients. In head and neck PET/CT protocol, some technical CT and PET parameters have been modified to cover the neck region and all regional lymph nodes. Furthermore, Terence el al. suggested that imaging quality improvements in the dedicated protocol may result in artifact elimination which could well define the adjacent anatomy (8).

Schlutter et al. performed ^18^F-FDG PET scan on 64 DTC patients with positive Tg and negative ^131^I WBS and illustrated that ^18^F-FDG PET was positive in 44 and negative in 20 patients (17). Overall, 34 true positive lesions were detected, which required surgery and/or external beam radiation therapy after ^18^F-FDG PET/CT scan in 19 out of 34 patients. Positive and negative predictive values of ^18^F-FDG PET/CT for detecting recurrence/metastasis were 83% and 25%, respectively. Also, Na et al. studied 54 DTC patients with negative ^131^I WBS, using off-T_4_, and ^18^F-FDG PET. In this study, the sensitivity and specificity of ^18^F-FDG PET in detecting recurrence/metastasis were 93.9% and 95.2%, respectively (18).

In the present study, 43 (62.3%) out of 69 DTC patients had positive ^18^F-FDG PET/CT results, meanwhile only 26 (37.7%) cases had positive CT results. The number of lesions detected on ^18^F-FDG PET/CT was more than twice the number detected on CT scan (92 lesions on PET/CT, compared to 39 lesions on CT scan). Therefore, ^18^F-FDG PET/CT was superior to CT scan in detecting recurrence and metastasis in DTC patients with high Tg level and negative ^131^I WBS.

^18^F-FDG PET/CT examination has the advantage of detecting both glucose metabolism in PET and abnormal anatomic changes in CT scan (19). In general, the criterion for suspecting metastatic cervical lymph nodes on CT scan was enlarged lymph nodes with the largest dimension greater than 10 mm (10)(20). However, in our study, 16 (37.2%) out of 43 patients had positive ^18^F-FDG PET lymph nodes less than 10 mm in diameter, whereas recurrence and metastasis were confirmed by post-operative histopathology in 10 patients. Consequently, there would be an underestimation if this criterion was taken into account for the interpretation of CT images.

The diagnostic accuracy of ^18^F-FDG PET/CT for recurrent and metastatic thyroid carcinoma is also related to increasing serum Tg level (21). The sensitivity, specificity, accuracy, positive predictive value and negative predictive value of ^18^F-FDG PET/CT were 69.4%, 66.7%, 69.1%, 95.6% and 17.4%, respectively. Therefore, the diagnostic accuracy of ^18^F-FDG PET/CT scan in DTC patients with positive Tg and negative ^131^I WBS may depend on serum Tg level at the time of imaging.

Overall, ^18^F-FDG PET/CT scan is useful for the detection and localization of malignant recurrences in patients with negative diagnostic radioiodine scan, despite elevated Tg level greater than 20 ng/ml or high anti-Tg level. However, ^18^F-FDG PET/CT provides little additional information when Tg level is lower than 5 ng/ml (18).

Among 31 patients with positive ^18^F-FDG PET/CT undergoing surgical resections, recurrence and metastasis were histopathologically confirmed in 30 patients, and only one patient had negative post-operative histopathological results. However, three patients with negative ^18^F-FDG PET/CT results had metastatic cervical lymph nodes during 6-12-month follow-up after ^18^F-FDG PET/CT scans. Therefore, ^18^F-FDG PET/CT results may be false positive due to FDG uptake in inflammatory lesions or false negative in case of microscopic metastatic diseases below the resolution of PET.

Principally, it is important that ^18^F-FDG PET/CT results be accompanied by patient’s clinical characteristics, other diagnostic tools as well as close follow-up in DTC patients using serum Tg and neck ultrasound(22). Undoubtedly, ^18^F-FDG PET/CT is more powerful than CT alone in detecting recurrent and metastatic lesions in DTC patients with high Tg level and negative ^131^I WBS.

On the other hand, ^18^F-FDG PET/CT is costly and unavailable in many nuclear medicine departments in developing countries such as Vietnam. As a result, the combination of conventional imaging modalities such as neck ultrasound and Tc-99m sestamibi SPECT/CT can be a reasonable approach for DTC patients with high Tg level and negative ^131^I WBS in hospitals not equipped with a PET/CT unit.

In the present study, after performing ^18^F-FDG PET/CT scan, 31 patients underwent surgical resections and two cases received external beam radiation therapy. Therefore, the treatment strategy changed in 33 out of 69 patients (47.8%), based on the results of ^18^F-FDG PET/CT imaging. In a study by Nahas et al., the treatment plan changed in 40% of patients, based on the FDG-PET/CT data (23).

The ability of FDG-PET/CT in detection of recurrent and metastatic lesions may affect the clinical results. As more positive lesions are detected, more patients are considered to require changes in the treatment strategies. On the other hand, in a group of patients, who cannot be treated by surgery or radiation therapy, other strategies should be considered. Empiric ^131^I therapy may be a suitable modality for these patients. Kuang et al. revealed that Tg level decreased in 63% of DTC patients after empiric ^131^I therapy (24). As the authors suggested, empiric therapy may be effective when Tg level is considered as an index of tumor burden.

## Conclusion

As the results indicated, the dedicated head and neck ^18^F-FDG PET/CT protocol was useful for the detection and localization of recurrent and/or metastatic lesions in post-surgical DTC patients with a high serum Tg level and negative ^131^I WBS. We recommend that treatment decision-making for these patients be based on ^18^F-FDG PET/CT results.

## References

[1] Bảo PTM, Hà LN (2006). Experiences of I(131) therapy in differentiated thyroid carcinoma. Clin Med Oncol.

[2] Mazzaferri EL, Jhiang SM (1994). Long-term impact of initial surgical and medical therapy on papillary and follicular thyroid cancer. Am J Med.

[3] Ha LN, Nhung NT, Son MH, Bieu BQ (2014). Clinical characteristics and preliminary evaluation of empirical 131 I therapy in differentiated thyroid carcinoma patients with negative 131 I whole? Body scan and elevated serum thyroglobulin. J Clin Med Pharma.

[4] Cooper DS, Doherty GM, Haugen BR, Kloos RT, Lee SL, Mandel SJ (2009). Revised American Thyroid Association management guidelines for patients with thyroid nodules and differentiated thyroid cancer. Thyroid.

[5] Townsend DW, Cherry SR (2001). Combining anatomy and function: the path to true image fusion. Eur Radiol.

[6] Blodgett TM, Ryan A, Akbarpouranbadr A, McCook BM (2007). PET/CT protocols and artifacts in the head and neck. PET Clin.

[7] Boellaard R, O’Doherty MJ, Weber WA, Mottaghy FM, Lonsdale MN, Stroobants SG (2010). FDG PET and PET/CT: EANM procedure guidelines for tumour PET imaging: version 1.0. Eur J Nucl Med Mol Imaging.

[8] Wong TZ, Fras IM (2007). PET/CT protocols and practical issues for the evaluation of patients with head and neck cancer. PET Clin.

[9] Fleming ID, Cooper JS, Henson DE, Hutter VP, Kennedy BJ, Murphy GP (2010). American joint committee on cancer. AJCC Cancer Staging Manual.

[10] Som PM (1992). Detection of metastasis in cervical lymph nodes: CT and MR criteria and differential diagnosis. AJR Am J Roentgenol.

[11] Bannas P, Derlin T, Groth M, Apostolova I, Adam G, Mester J (2012). Can (18)F-FDG-PET/CT be generally recommended in patients with differentiated thyroid carcinoma and elevated thyroglobulin levels but negative I-131 whole body scan?. Ann Nucl Med.

[12] Shammas A, Degirmenci B, Mountz JM, McCook BM, Branstetter B, Bencherif B (2007). 18F-FDG PET/CT in patients with suspected recurrent or metastatic well-differentiated thyroid cancer. J Nucl Med.

[13] Marcus C, Whitworth PW, Surasi DS, Pai SI, Subramaniam RM (2014). PET/CT in the management of thyroid cancers. AJR Am J Roentgenol.

[14] Feine U, Lietzenmayer R, Hanke JP, Wohrle H, Muller-Schauenburg W (1995). [18FDG whole-body PET in differentiated thyroid carcinoma. Flipflop in uptake patterns of 18FDG and 131I]. Nuklearmedizin.

[15] Yamamoto Y, Wong TZ, Turkington TG, Hawk TC, Coleman RE (2007). Head and neck cancer: dedicated FDG PET/CT protocol for detection--phantom and initial clinical studies. Radiology.

[16] Beyer T, Antoch G, Muller S, Egelhof T, Freudenberg LS, Debatin J (2004). Acquisition protocol considerations for combined PET/CT imaging. J Nucl Med.

[17] Schluter B, Bohuslavizki KH, Beyer W, Plotkin M, Buchert R, Clausen M (2001). Impact of FDG PET on patients with differentiated thyroid cancer who present with elevated thyroglobulin and negative 131I scan. J Nucl Med.

[18] Na SJ, Yoo IeR, O JH, Lin C, Lin Q, Kim SH (2012). Diagnostic accuracy of (18)F-fluorodeoxyglucose positron emission tomography/computed tomography in differentiated thyroid cancer patients with elevated thyroglobulin and negative (131)I whole body scan: evaluation by thyroglobulin level. Ann Nucl Med.

[19] Townsend DW (2008). Dual-modality imaging: combining anatomy and function. J Nucl Med.

[20] van den Brekel MW, Stel HV, Castelijns JA, Nauta JJ, van der Waal I, Valk J (1990). Cervical lymph node metastasis: assessment of radiologic criteria. Radiology.

[21] Bertagna F, Bosio G, Biasiotto G, Rodella C, Puta E, Gabanelli S (2009). F-18 FDG-PET/CT evaluation of patients with differentiated thyroid cancer with negative I-131 total body scan and high thyroglobulin level. Clin Nucl Med.

[22] Rosario PW, de Faria S, Bicalho L, Alves MF, Borges MA, Purisch S (2005). Ultrasonographic differentiation between metastatic and benign lymph nodes in patients with papillary thyroid carcinoma. J Ultrasound Med.

[23] Nahas Z, Goldenberg D, Fakhry C, Ewertz M, Zeiger M, Ladenson PW (2005). The role of positron emission tomography/computed tomography in the management of recurrent papillary thyroid carcinoma. Laryngoscope.

[24] Ma C, Xie J, Kuang A (2005). Is empiric 131I therapy justified for patients with positive thyroglobulin and negative 131I whole-body scanning results?. J Nucl Med.

